# Author Correction: The temporal dynamics and infectiousness of subpatent *Plasmodium falciparum* infections in relation to parasite density

**DOI:** 10.1038/s41467-019-10790-0

**Published:** 2019-06-11

**Authors:** Hannah C. Slater, Amanda Ross, Ingrid Felger, Natalie E. Hofmann, Leanne Robinson, Jackie Cook, Bronner P. Gonçalves, Anders Björkman, Andre Lin Ouedraogo, Ulrika Morris, Mwinyi Msellem, Cristian Koepfli, Ivo Mueller, Fitsum Tadesse, Endalamaw Gadisa, Smita Das, Gonzalo Domingo, Melissa Kapulu, Janet Midega, Seth Owusu-Agyei, Cécile Nabet, Renaud Piarroux, Ogobara Doumbo, Safiatou Niare Doumbo, Kwadwo Koram, Naomi Lucchi, Venkatachalam Udhayakumar, Jacklin Mosha, Alfred Tiono, Daniel Chandramohan, Roly Gosling, Felista Mwingira, Robert Sauerwein, Richard Paul, Eleanor M Riley, Nicholas J White, Francois Nosten, Mallika Imwong, Teun Bousema, Chris Drakeley, Lucy C Okell

**Affiliations:** 1 MRC Centre for Global Infectious Disease Analysis, Department of Infectious Disease Epidemiology, Imperial College London, London, W2 1PG UK; 2 Department of Epidemiology and Public Health, Swiss Tropical and Public Health Institute, Basel, 4002 Switzerland; 3 University of Basel, Basel, 4001 Switzerland; 4 Medical Parasitology and Infection Biology, Swiss Tropical and Public Health Institute, Basel, 4002 Switzerland; 5 Vector-borne Diseases Unit, Papua New Guinea Institute for Medical Research, Madang, Papua New Guinea; 6 Division of Population Health and Immunity, The Walter and Eliza Hall Institute of Medical Research, Parkville, 3052 VIC Australia; 7 Medical Biology, University of Melbourne, Melbourne, 3010 VIC Australia; 8 Disease Elimination, Burnet Institute, Melbourne, 3004 VIC Australia; 9 MRC Tropical Epidemiology Group, London School of Hygiene and Tropical Medicine, London, WC1E 7HT UK; 10 Department of Immunology and Infection, London School of Hygiene and Tropical Medicine, London, WC1E 7HT UK; 11 Malaria Research, Department of Microbiology, Tumor and Cell Biology, Karolinska Institutet, 171 77, Stockholm, Sweden; 12 Département de Sciences Biomédicales, Centre National de Recherche et de Formation sur le Paludisme, Ouagadougou, 01 BP 2208 Burkina Faso; 13 Institute for Disease Modeling, Intellectual Ventures, Bellevue, 98005 Washington USA; 14 Department of Training and Research, Mnazi Mmoja Hospital, Zanzibar, Tanzania; 15 Population Health and Immunity Division, Walter and Eliza Hall Institute, Melbourne, 3052 Victoria Australia; 16 Department of Biological Sciences, University of Notre Dame, Indiana, 46556 USA; 17 Department of Parasites and Insect Vectors, Institut Pasteur, Paris, 75015 France; 18 Medical Biology, University of Melbourne, Melbourne, 3010 VIC Australia; 19 Radboud Institute for Health Sciences, Radboud University Medical Centre, Nijmegen, 6525 The Netherlands; 20 Armauer Hansen Research Institute, Addis Ababa, Ethiopia; 21 Institute of Biotechnology, Addis Ababa University, Addis Ababa, Ethiopia; 22 Diagnostics Program, PATH, Seattle, Washington, 98121 United States of America; 23 Centre for Tropical Medicine and Global Health, Nuffield Department of Medicine, University of Oxford, Oxford, OX3 7FZ UK; 24 KEMRI–Wellcome Trust Research Programme, Centre for Geographic Medicine Research-Coast, Kilifi, Kenya, Centre for Genomics and Global Health, Wellcome Trust Centre for Human Genetics, University of Oxford, Oxford, OX3 7BN UK; 25 Institute of Health, University of Health and Allied Sciences, Hohoe, PMB 31 Ghana; 26 Sorbonne Université, INSERM, Institut Pierre-Louis d’Epidémiologie et de Santé Publique, AP- HP, Groupe Hospitalier Pitié-Salpêtrière, Service de Parasitologie-Mycologie, Paris, 75646 France; 27 Malaria Research and Training Centre, Parasitic Diseases Epidemiology Department, UMI 3189, University of Sciences, Technique and Technology, Bamako, Mali; 28 Noguchi Memorial Institute for Medical Research, University of Ghana, Legon, Ghana; 29 Malaria Branch, Division of Parasitic Diseases and Malaria, Centers for Global Health, Centers for Disease Control and Prevention, Atlanta, 30030 GA United States of America; 30 National Institute for Medical Research, Mwanza Medical Research Centre, Mwanza, Tanzania; 31 Department of Biomedical Sciences, Centre National de Recherche et de Formation sur le Paludisme, Ouagadougou, 01 BP 2208 Burkina Faso; 32 Department of Disease Control, London School of Hygiene and Tropical Medicine, Keppel Street, London, WC1E 7HT UK; 33 Malaria Elimination Initiative, Global Health Group, University of California, San Francisco, San Francisco, 94158 CA United States; 34 Biological Sciences Department, Dar es Salaam University College of Education, P. O. Box 2329, Dar es Salaam, Tanzania; 35 Institut Pasteur de Dakar, Laboratoire d‘Entomologie Médicale, Dakar, Senegal; 36 The Roslin Institute and Royal (Dick) School of Veterinary Studies, University of Edinburgh, Easter Bush, Midlothian, EH25 9RG UK; 37 Centre for Tropical Medicine and Global Health, Nuffield Department of Clinical Medicine, University of Oxford, Oxford, OX3 7FZ UK; 38 Mahidol Oxford Tropical Medicine Research Unit, Faculty of Tropical Medicine, Mahidol University, Bangkok, 10400 Thailand; 39 Shoklo Malaria Research Unit, Mahidol–Oxford Tropical Medicine Research Unit, Faculty of Tropical Medicine, Mahidol University, Mae Sot, 63110 Thailand; 40 Department of Molecular Tropical Medicine and Genetics, Faculty of Tropical Medicine, Mahidol University, Bangkok, 10400 Thailand

**Keywords:** Malaria, Diagnosis

Correction to: *Nature Communications* 10.1038/s41467-019-09441-1; published online 29 March 2019

The original version of this Article omitted Richard Paul, who is from the ‘Institut Pasteur de Dakar, Laboratoire d'Entomologie Médicale, Dakar, Senegal.’ The following statement has been added to the Acknowledgements: ‘R.Pa. would like to acknowledge funding from the Strategic Anopheles Horizontal Research Programme, Institut Pasteur.’ Additionally, ‘R.Pa.’ has been added to the third sentence of the Contributions, and ‘R.P.’ has been updated to ‘R.Pi’: ‘I.F., N.H., L.R., J.C., B.P.G., A.B., A.L.O., U.M., M.M., C.K., I.M., F.T., E.G., S.M., G.D., M.K., J.Mi., S.O.A., C.N., R.Pi., O.D., S.N.D., K.K., N.L., V.U., J.Mo., A.T., D.C., R.G., F.M., R.S., R.Pa., E.M.R., N.J.W., F.N., M.I., T.B., and C.D. collected the data.’ These changes have now been made in both the HTML and PDF version of the Article.

