# Supplementary material for: The temporal dynamics and infectiousness of subpatent Plasmodium falciparum infections in relation to parasite density
Source: Nat Commun. 2019 Mar 29;10:1433. doi: 10.1038/s41467-019-09441-1 (PMC6440965; doi:10.1038/s41467-019-09441-1)
Supplement: Supplementary file 1 — Supplementary Information [file 41467_2019_9441_MOESM1_ESM.docx]

**Supplementary Table 1: Details of datasets used for figures 1 and 2**

Studies measuring subpatent densities using a quantitative molecular method. NB none of these studies reported copy numbers only.

| Location and study name | Year(s) of data collection | Age of participantS | AUTHOR | Reference | Molecular method for assessing densities | Details of molecular method (target, blood volume, target volume) | % positive by molecular method | N tested by molecular method | Standard diagnostic used | % standard diagnostic positive |
| --- | --- | --- | --- | --- | --- | --- | --- | --- | --- | --- |
| Papua New Guinea | 2010 | All age | Koepfli | ^1^ | Genus specific qPCR | 18S rRNA, 200µL blood, ? | 18.5% | 2083 | Microscopy | 5.6% |
| Burkina Faso (NUFFIC) | 2007 - 2008 | All age | Ouedraogo | ^2^ | QT-NASBA | 18S rRNA, 100µL blood, ? | 90.6% | 307 | Microscopy | 40.1% |
| Uganda BIOME | 2015 | Children aged 0.5 - 11 & primary caregivers | Das | ^3^ | qRT-PCR | 18S rRNA, 100µL blood, 2mL | 43.0% | 607 | Microscopy, RDT and HS-RDT | 20.1% 29.4% 41% |
| Myanmar SMRU/ BIOME | 2015 | All ages | Das | ^3^ | qRT-PCR | 18S rRNA, 50µL blood, 2ml | 1.8% | 493 | RDT and HS-RDT | 0%  1% |
| Tanzania | 2013 | All age | Hofmann | ^4^ | qPCR TARE-2 varATS | 18S rRNA, telomere-associated repetitive element 2, var gene acidic terminal sequence, ?,? | 49.1% 57.7% 57.3% | 503 | Microscopy | 25.0% |
| Thai-Myanmar border & Cambodia | 2013 | All age | Imwong | ^5^ | Absolute quantitative real time PCR | 18S rRNA, 100-2000µL blood, 2mL | 0.8% (Pailin) 0.61% (T-M border) | 7231  9431 | NA | NA |
| China | 2013 | All age | Cheng | ^6^ | CLIP-PCR qPCR | 18s rRNA, , 3mm punch of dried blood spot or 60 µl,? | 0.4% | 17 | Microscopy and RDT | 0.3% 0.4% |
| Ethiopia | 2015 | 12 (IQR 11–14) | Tadesse | ^7^ | qPCR | 18s rRNA, 100µl cell pellet, ? | 9.7% | 845 | Microscopy | 0.9% |
| Kenya and Burkina Faso (AFIRM) | 2013-2014 | All ages | Goncalves | ^8^ | qPCR and QT-NASBA | 18S rRNA, 100µL | 66.5% (BF) 38.0% (E Kenya) | 400 413 | Microscopy | 38.8% 13.6% |
| Location and study name | **Year(s) of data collection** | **Age of participants** | **Author** | **Reference** | **Molecular method for assessing densities** | **Details of molecular method (target, blood volume, target volume)** | **% positive by molecular method** | **N tested by molecular method** | **Standard diagnostic used** | **% standard diagnostic positive** |
| Haiti | 2011 | All ages | Lucchi | ^9^ | PET-PCR | 18s rRNA, 3mm punch of dried blood spot, ?) | 0.4% | 12 | Microscopy and RDT | 0.3% 0.2% |
| Tanzania | 2010 | All ages | Mosha | ^10^ | qPCR | Met tRNA, ?,? | 35.3% | 3057 | RDT | 7.3% |
| Tanzania (ALIVE) | 2011 | All ages | Mwingira | ^11^ | qPCR | 18s rRNA, 50µl blood,  ? | 38.1% | 226 | Microscopy and RDT | 5.8% 15.9% |
| Mali | 2012-2013 | ? | Nabet | ^12^ | qPCR | 18s rRNA, ?,? | 19% | 648 | NA | NA |
| Zanzibar | 2005, 2011, 2013 | All ages | Morris | ^13^ | qPCR | cytb, 3-5 µl blood, ? | 26.4% (2005) 2.2% (2011) 2.3% (2013) | 534 4131 2977 | Microscopy (2005) and RDT (2011, 2013) | 7.5% 0.4% 0.3% |
| Zanzibar | 2015 | All ages | Aydin-Schmidt | ^14^ | qPCR | cytb, 3-5 µl blood, ? | 1.8% | 3013 | RDT | 0.4% |
| Zanzibar | 2013 | All ages | Cook | ^15^ | Real-time PCR | Chelex-100 method using one filter paper punch (3–5 μl), cytb | 2.1% (round 1)  2.6% (round 2) | 6233  4894 | RDT | 0.2%  0.2% |
| Ethiopia | 2016 | All ages | Tadesse | - | qPCR | 18s rRNA, ?, ? | 9.7% | 82 | Microscopy | 1.6% |

**Supplementary Table 2: Longitudinal studies in endemic areas with individual follow up by microscopy and a molecular method: further details (Table 1)**

| **COUNTRY** | **LOCATION** | **YEAR OF DATA COLLECTION** | **N** | **AGE OF PARTICIPANTS, YEARS** | **% SAMPLES SLIDE POSITIVE (n/N)** | **% SAMPLES POSITIVE BY MOLECULAR METHODS (n/N)** | **TREATMENT** | **TREATMENT RATE PER PERSON / 2 MONTH INTERVAL** | **PUBLICATION (FIRST AUTHOR, YEAR)** | **MOLECULAR METHOD (TARGET, VOLUME BLOOD, VOLUME TEMPLATE) NR = not reported** | **COMMENTS** |
| --- | --- | --- | --- | --- | --- | --- | --- | --- | --- | --- | --- |
| **SENEGAL** | Gouye Kouly | 2004  2005 | 369  169 | All ages | 14.0  (259/1848)  7.1  (199/2813) | 14.5  (269/1848)  10.1  (277/2738) | 23 treatments within 9 weeks.  11 treatments within 9 weeks | 0.06  0.06 | Lawaly 2012 ^16^ | PCR (ssrDNA, 100-300µl blood, NR) |  |
| **PAPUA NEW GUINEA** | Ilaita | 2006-2007 | 264 | 1-4.5 | 23 (737/3140) | 36  (1128/3140) | Median 6 treatments per child over 16 months, for *Pf* & *Pv* | 0.78 | Lin, Kiniboro 2010 ^17^, Koepfli 2011 ^18^ | LDR-FMA (ssrDNA, NR,NR) |  |
| **GHANA** | Southern | 1994-1996 | 143 | 0-1 | 20.9 (559/2670) | 28.1  (776/2760) | 89 cases of clinical malaria were treated  in 52 children over ~ 2 years ^19^ | 0.05 | Wagner 1998 ^20^ | PCR (7H8/6, NR, 1µl template) | Birth cohort |
| **SENEGAL** | Thies | Oct 2003 | 21 | 5-15 | 100  (168/168) | 100  (168/168) | No treatment | 0 | Jafari-Guemouri 2006 ^21^ | PCR (msp2, 200µl blood, NR) | Slide-positive at baseline |
| **GHANA** | Kassena-Nankana | 2000-2001 | 100 | All ages | 56 (1042/1851) | 88  (1436/1851) | No data | - | Owusu-Agyei 2002 ^22^ | PCR-RFLP & GeneScan (msp2, 10µl blood, NR) |  |
| **TANZANIA** | Kiberge | 1996 | 60 | 0-2 | 65  (267/419) | 82  (338/410) | Chloroquine detected in 25% urine samples | - | Fraser-Hurt 1999 ^23^ | PCR (msp2, NR,NR) |  |

**Supplementary Table 3: Details of studies used for figure 6 (gametocytaemia)**

| Location and study name | Year(s) of data collection | Age of participants, years (range) | Publication (1^st^ author, year) | Citation | Molecular method for assessing densities | Details of molecular method (target, blood volume) | % Gametocyte positive | N tested by molecular method |
| --- | --- | --- | --- | --- | --- | --- | --- | --- |
| Papua New Guinea | 2010 | All age | Koepfli | ^1^ | qRT-PCR | Pfs25 rRNA, 200µL blood | 11.2% | 2083 |
| Burkina Faso (NUFFIC) | 2007 - 2008 | All age | Ouedraogo | ^2^ | QT-NASBA | Pfs25 rRNA, 100µL blood | 67.4% | 307 |
| Kenya and Burkina Faso (AFIRM) | 2013-2014 | All ages | Goncalves | ^8^ | mRNA QT-NASBA | Pfs25 rRNA, 100µL | 61.8% (BF) 25.7% (E Kenya) | 400  413 |
| Tanzania (ALIVE) | 2011 | All ages | Mwingira | ^11^ | qRT-PCR | Pfs25 rRNA, 50µl | 10.6% | 226 |
| Ethiopia | 2016 | All ages | Tadesse | ^23^ | qRT-PCR | Pfs25 rRNA, 100µL | 1.5% | 882 |
| BURKINA FASO | 2005 | Children <14 years | Ouedraogo | ^24^ | QT-NASBA | Pfs25, 3ml (for membrane feeding and QT-NASBA) | 70.1% | 412 |

**Supplementary Table 4: Details of studies used for figures 7 and 8 (membrane feeding studies)**

| Location and study name | Year(s) of data collection | Age of participants, years (range) | author | Citation | mosquito species | Feeding method | infectivity enedpoint |
| --- | --- | --- | --- | --- | --- | --- | --- |
| Burkina Faso (NUFFIC) | 2007 - 2008 | All age | Ouedraogo | ^2^ | An. gambiae | Membrane | Oocysts |
| EAST Kenya (AFIRM) | 2013-2014 | All ages | Goncalves | ^8^ | An. gambiae | Membrane | Oocysts |
| West kenya (afirm) | 2013-2014 | All ages | Goncalves | ^8^ | An. gambiae | Membrane | Oocysts |
| burkin faso (afrim) | 2013-2014 | All ages | Goncalves | ^8^ | An. gambiae | Membrane | Oocysts |
| senegal | 2011 | >5 year olds | Gaye | ^25^ | An. arabiensis | Direct | Oocysts |
| West Kenya | 1991 | All ages | Githeko | ^26^ | An. gambiae | Direct | Oocysts & CSP ELISA |
| Ethiopia | 2016 | All ages | Tadesse | ^23^ | An. arabiensis | Membrane | CSP ELISA and Pfs25 qPCR |

**Supplementary Figure 1: Ratio of sensitivity of microscopy in high and low transmission season**


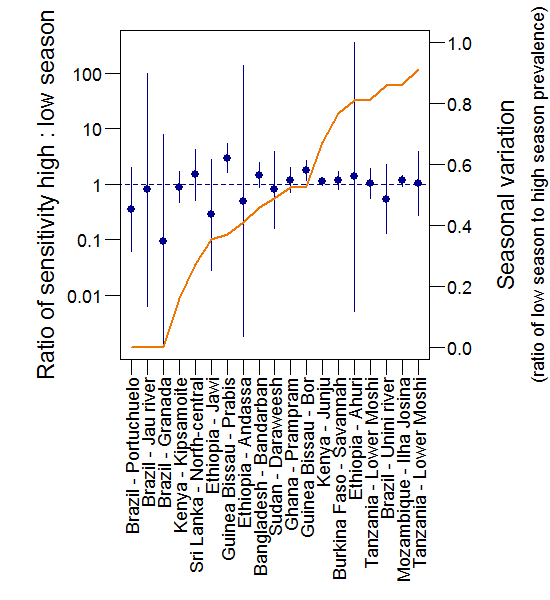


*Supplementary Figure 1: Difference in sensitivity of microscopy relative to PCR as gold standard in different seasons. Circles show the ratio of the sensitivity in the high transmission season to the sensitivity in the dry season in locations with 2 or more surveys, with 95% CI. A ratio greater than 1 indicates greater sensitivity of microscopy in the high transmission (rainy) season versus the low transmission season. Wide 95% CI are seen on some of these ratios usually due to small numbers testing positive in low transmission sites. Sites are ordered according to the degree of seasonal variation in slide prevalence, with the most seasonally varying sites on the left. We calculated seasonal variation as the slide-prevalence in the low transmission season divided by the slide-prevalence in the high transmission season in the same area (orange line). For 2 locations in Ethiopia where slide prevalence was zero in both seasons, we used PCR prevalence to calculate seasonal variation.*

**Supplementary Figure 2: Differences in the parasite densities of submicroscopic vs. sub-RDT infected individuals**

Four studies used both microscopy and RDT. We compared the qPCR parasite densities of individuals with submicroscopic vs. sub-RDT infections for each of these studies. Data from China and Haiti are just presented for visual purposes due to the small sample sizes. We performed a Kolmogorov-Smirnoff two-sample test on the data from Uganda and Tanzania to assess the equality of the submicroscopic and sub-RDT parasite density distributions for each study. The distributions were found to not be significantly different (p>0.05).


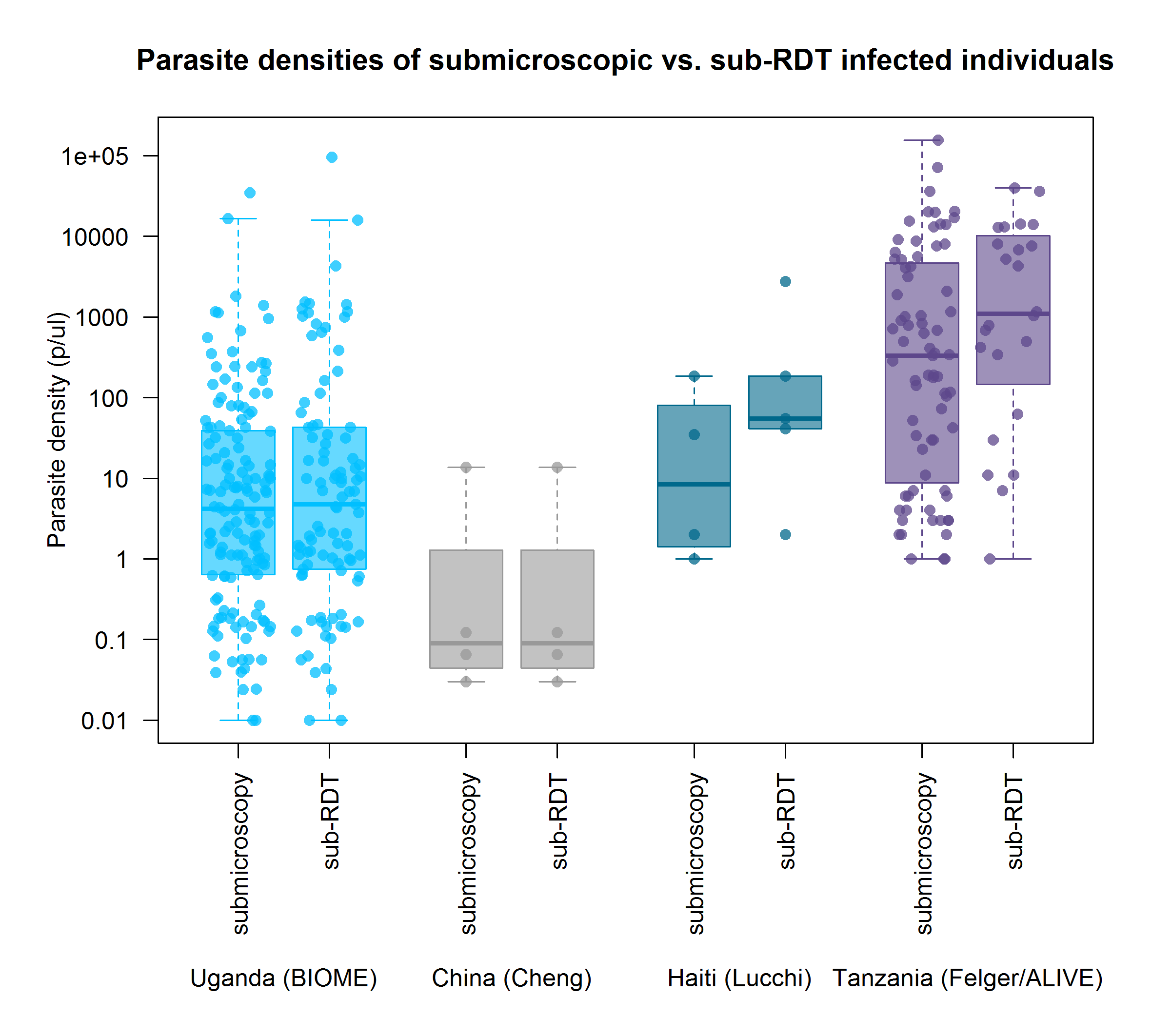


*Supplementary Figure 2: Quantitative PCR parasite densities of all individuals with submicroscopic and sub-RDT infection (in many cases, individuals had both sub-RDT and submicroscopic infections).*

**Supplementary Note 2: Impact on non-standardised age profiles in datasets**

All the datasets have different proportions of individuals in different age groups. Here we standardise all datasets that contain age information (17/22) to investigate the impact of different age profiles on the results. Data are now weighted such that, for each dataset, 15% of the population are 5 years old or younger, 35% of the population are between 6 and 15, and 50% of the population are aged 16 or older.

The age-standardised geometric mean parasite densities were calculated by calculating the geometric mean for each age group within each study, denoted by $d_{i}$. The relative weights $w$ for age group $i$ were then calculated as:

$w_{i}=\theta_{i}/(n_{i}/n)$ [1]

Where $\theta_{i}$ is the desired weight of each age group (0.15, 0.35 or 0.5), $n_{i}$ is the number of people in age group $i$ and $n$ is the total number of people in all the age groups. Finally the age group standardised geometric mean is then calculated as:

$M= {10}^{^}\left( \frac{\sum_{i} \log_{10} \left( d_{i} \right)w_{i}}{\sum_{i} w_{i}} \right)$ [2]

The weighted boxplot was produced using the ‘wtd.boxplot’ function in the package ‘ENmisc’ in R.


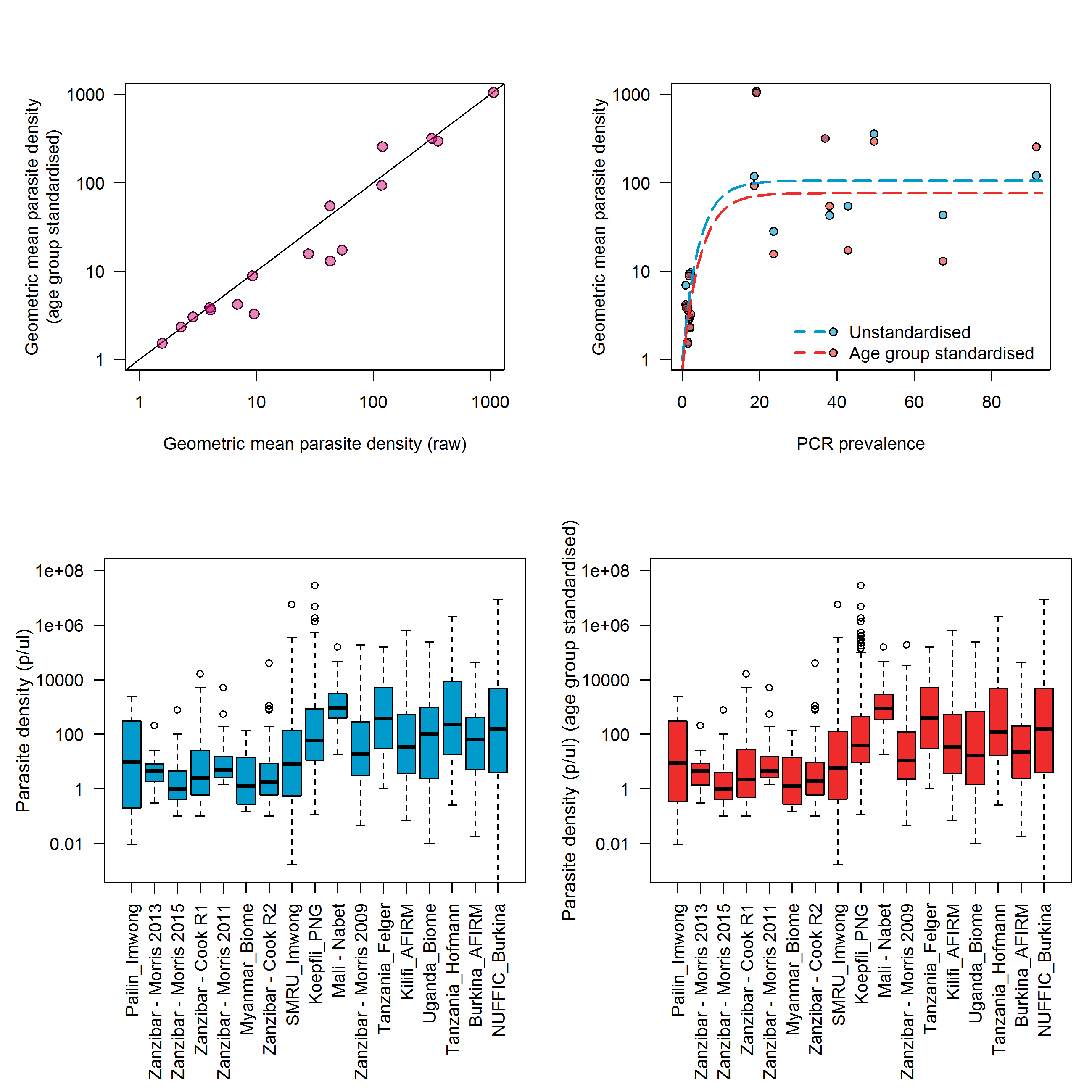


*Supplementary Figure 3:. A) Comparison of the geometric mean parasite density for each study with individual level age data unstandardised vs. standardised. B) Geometric mean parasite density for each study plotted against survey-level PCR prevalence for unstandardised and standardised data. C) and D) boxplots of unstandardised (blue) and standardised (red) parasite density distributions from each study*

There is no significant difference between the geometric mean parasite densities of the unstandardised and standardised data sets (p>0.05 using paired t-test). The plateau geometric mean parasite density in moderate and high transmission settings (Figure S3B) is 105 parasites per µl and 76 parasites per µl for the unstandardised and standardised data respectively.

**Supplementary Note 2: Details of model fitting and parameter values from Figure 2**

A simple exponential model of the form $y=a-b exp(mx)$ was fitted to the proportions of PCR positive individuals with parasites densities above the three limits of detection (100, 10 and 1 parasite per µl) using weighted (based on the number of samples from each study) nonlinear least squares regression. Table S2.1 presents the resulting parameter estimates.

| Limit of detection | a | b | m |
| --- | --- | --- | --- |
| 1 parasites per µl | 0.9319 | 0.3435 | -15.4420 |
| 10 parasites per µl | 0.6377 | 0.4188 | -8.2792 |
| 100 parasites per µl | 0.4912 | 0.4508 | -6.5870 |

**Supplementary Methods 1: Details of calculations used to estimate the relative infectivity to mosquiotoes of patent compared to subpatent individuals (Figure 8)**

***Methodology used to weight data***

For seven out of eight of the datasets, we have data on the age of each individual (no age data for Coleman et al.). The data are weighted such that each age group has a consistent age distribution (15% of the population ≤5, 35% of the population >5 and ≤15, and 50% of the population >15. Each individuals in an age group is then weighted based on their propensity to receive bites and use a bed net. All values for age group proportions and weights are taken from Stone et al. ^27^

| Age group | Group number | Proportion of population ($p_{Ai})$ | Biting weight ($w_{Bi})$ | Net use weight  ($w_{Ni})$ |
| --- | --- | --- | --- | --- |
| ≤5 | 1 | 0.15 | 1/8 | 1/2.9 |
| >5 and ≤15 | 2 | 0.3 | 3/8 | 1.2/2.9 |
| >15 | 3 | 0.55 | 4/8 | 0.7/2.9 |

Here we denote the desired proportion of the population in each age group as ($p_{A1},p_{A2},p_{A3})$, the biting weights as ($w_{B1},w_{B2},w_{B3})$ and the net use weights as ($w_{N1},w_{N2},w_{N3})$. We also denote the set of all individuals in each age group as $\{X_{1}\} ADDIN EN.CITE <EndNote><Cite><Author>!!! INVALID CITATION !!! </Author><RecNum>0</RecNum><DisplayText><style face="superscript">28</style></DisplayText><record><dates><year>!!! INVALID CITATION !!! </year></dates></record></Cite></EndNote>$, $\{X_{2}\}$,$\{X_{3}\}$. The number of individuals in each age group in the data (the cardinality of the set) is denoted as $|\left\{ X_{i} \right\}|$ where $i \epsilon\{1,2,3\}$.

i) Weighting by age group size (i.e. standardising age distribution between datasets)

Each individual in group $X_{i}$ is assigned a weight given by the following equation:

$z_{Ai=}\frac{\left| X_{i} \right|}{\sum_{i} |X_{i}|}$ [3]

ii) Weighting by biting weight and net use weight

Each individual in group $X_{i}$ is then assigned a weight to account for biting and net use given by the following equation:

$z_{B,Ni}=w_{Bi}w_{Ni}/\sum_{i} (w_{Bi}w_{Ni})$ [4]

Finally, these two weights are combined to get a final weight for group $i$: $z_{i}=z_{Ai}z_{B,Ni}$. These weights are then used when summing the number of mosquitoes infected and dissected by patent and subpatent individuals.

We also present here another version of Figure 8 where we have only standardised to get a matching age distribution, but have not weighted based on age-based biting exposure and net use (Figure S4). Here the overall estimate is 3.36 (95%CI: 2.95-3.84), which is slightly higher than the value presented in the main text (2.88 (95%CI: 2.54-3.26)).


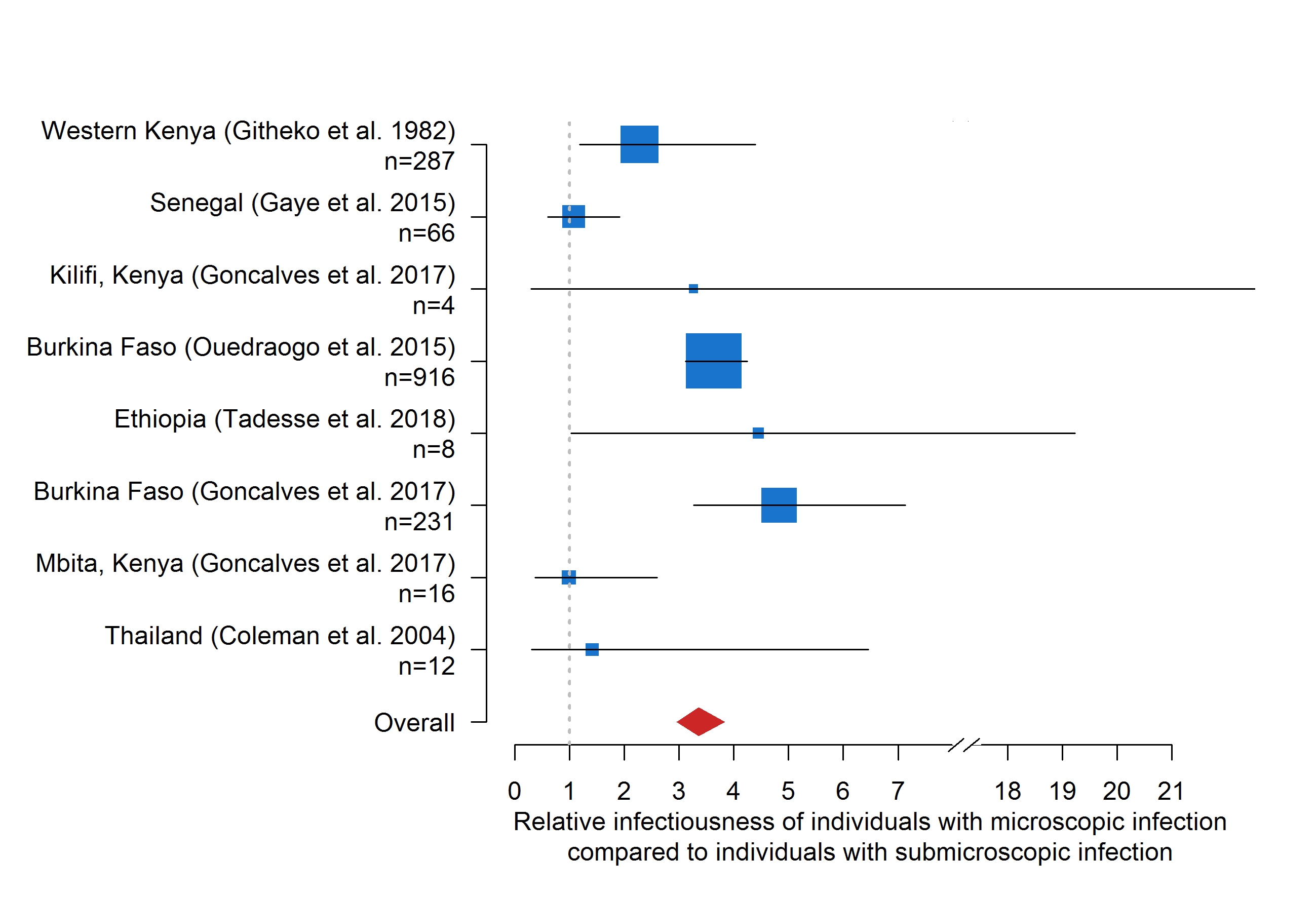


*Supplementary Figure 4: Relative infectiousness of patent compared to subpatent individuals standardising to ensure a consistent age distribution in each dataset, but not account for age-based differences in biting exposure and net usage.*

**Supplementary Methods 2:  *Methodology used to estimate subpatent infectivity in datasets with no PCR data***

In several of the datasets used in Figure 8, there is no PCR data to inform the ‘true’ subpatent (microscopy negative PCR positive) individual ^25,26,28^ and in one study there were no PCR data from one of the three sites (Mbita, Kenya)^29^.

We adopted a simple assumption to enable us to use these datasets in the analysis: for each study, we calculated the microscopy prevalence in each of the age groups, then used age-specific microscopy-PCR prevalence conversion curves from Okell et al.^30^ to estimate the PCR prevalence in each age group (Figure S5). We then assume that a proportion of all individuals that were negative by microscopy would have been positive by PCR based on these estimates. Further to this, we assume that all microscopy negative individuals that infected mosquitoes would be in this PCR positive, microscopy negative group. This is consistent with the datasets where we do have PCR data available: only 1 out of 383 PCR-, microscopy- indiviudals infected mosquitoes in Kilifi and Burkina Faso^29^ and 1 out of 307 PCR-, microscopy- indiviudals infected mosquitoes in another study in Burkina Faso^2^.


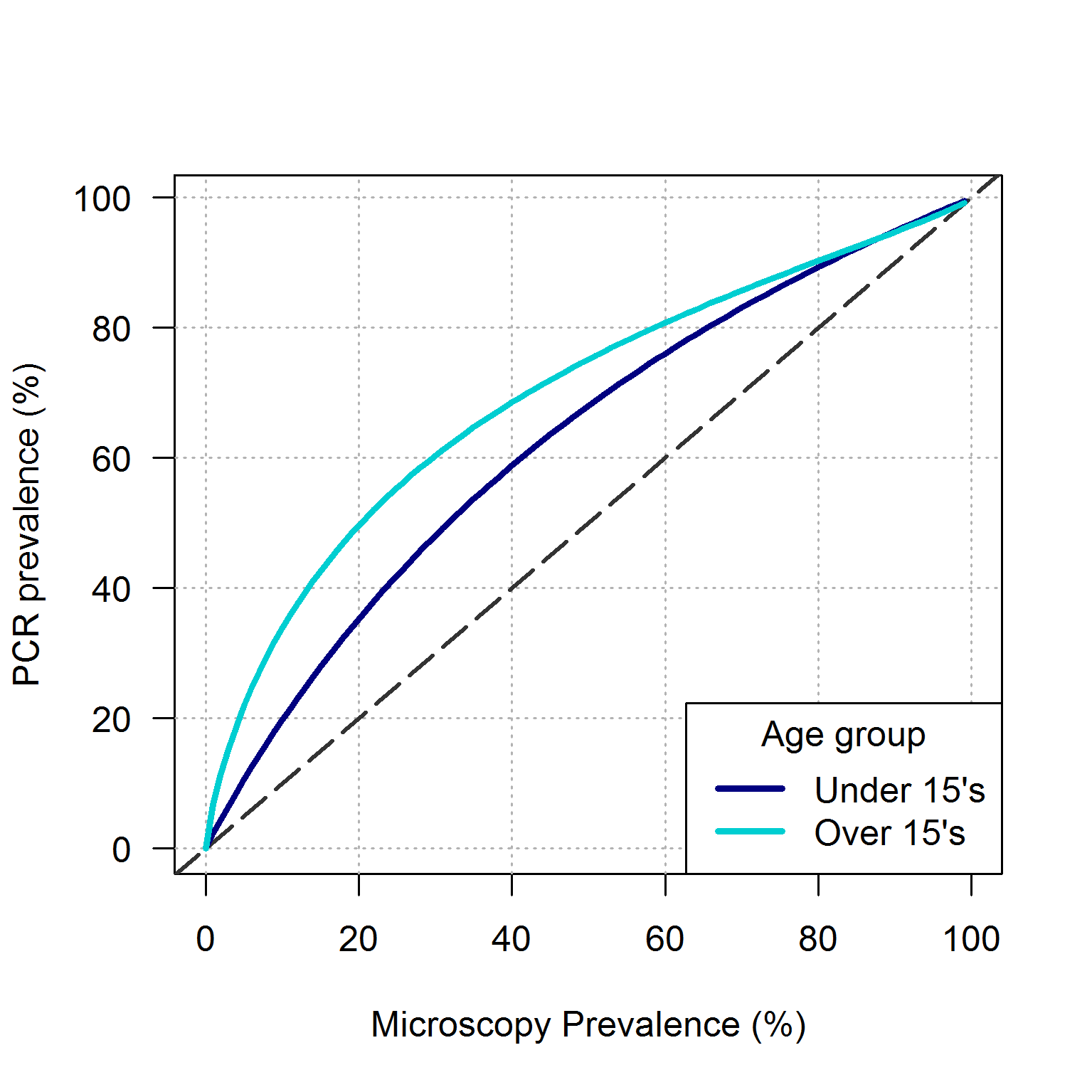


*Supplementary Figure 5: Microscopy-PCR prevalence conversion relationships^30^used to estimate the proportion of microscopy negative individuals that would be expected to be PCR positive.*

**References**

1 Koepfli, C. *et al.* Blood-Stage Parasitaemia and Age Determine Plasmodium falciparum and P. vivax Gametocytaemia in Papua New Guinea. *PLOS ONE* **10**, e0126747, doi:10.1371/journal.pone.0126747 (2015).

2 Ouedraogo, A. L. *et al.* Dynamics of the Human Infectious Reservoir for Malaria Determined by Mosquito Feeding Assays and Ultrasensitive Malaria Diagnosis in Burkina Faso. *J Infect Dis* **213**, 90-99, doi:10.1093/infdis/jiv370 (2016).

3 Das, S. *et al.* Performance of a high-sensitivity rapid diagnostic test for Plasmodium falciparum malaria in asymptomatic individuals from Uganda and Myanmar and naïve human challenge infections. Unpublished manuscript.

4 Hofmann, N. *et al.* Ultra-sensitive detection of Plasmodium falciparum by amplification of multi-copy subtelomeric targets. *PLoS Med* **12**, e1001788, doi:10.1371/journal.pmed.1001788 (2015).

5 Imwong, M. *et al.* Numerical Distributions of Parasite Densities During Asymptomatic Malaria. *J Infect Dis* **213**, 1322-1329, doi:10.1093/infdis/jiv596 (2016).

6 Cheng, Z. *et al.* Capture and Ligation Probe-PCR (CLIP-PCR) for Molecular Screening, with Application to Active Malaria Surveillance for Elimination. *Clin Chem* **61**, 821-828, doi:10.1373/clinchem.2014.237115 (2015).

7 Tadesse, F. G. *et al.* The shape of the iceberg: quantification of submicroscopic Plasmodium falciparum and Plasmodium vivax parasitaemia and gametocytaemia in five low endemic settings in Ethiopia. *Malar J* **16**, 99, doi:10.1186/s12936-017-1749-4 (2017).

8 Gonçalves, B. *et al.* The human infectious reservoir for Plasmodium falciparum malaria in areas of differing transmission intensity (unpublished).

9 Lucchi, N. W. *et al.* PET-PCR method for the molecular detection of malaria parasites in a national malaria surveillance study in Haiti, 2011. *Malar J* **13**, 462, doi:10.1186/1475-2875-13-462 (2014).

10 Mosha, J. F. *et al.* Epidemiology of subpatent Plasmodium falciparum infection: implications for detection of hotspots with imperfect diagnostics. *Malar J* **12**, 221, doi:10.1186/1475-2875-12-221 (2013).

11 Mwingira, F., Genton, B., Kabanywanyi, A. N. & Felger, I. Comparison of detection methods to estimate asexual Plasmodium falciparum parasite prevalence and gametocyte carriage in a community survey in Tanzania. *Malar J* **13**, 433, doi:10.1186/1475-2875-13-433 (2014).

12 Nabet, C. *et al.* Genetic diversity of Plasmodium falciparum in human malaria cases in Mali. *Malar J* **15**, 353, doi:10.1186/s12936-016-1397-0 (2016).

13 Morris, U. *et al.* Characterising temporal trends in asymptomatic Plasmodium infections and transporter polymorphisms during transition from high to low transmission in Zanzibar, 2005-2013. *Infect Genet Evol* **33**, 110-117, doi:10.1016/j.meegid.2015.04.018 (2015).

14 Aydin-Schmidt, B. *et al.* Loop mediated isothermal amplification (LAMP) accurately detects malaria DNA from filter paper blood samples of low density parasitaemias. *PLoS One* **9**, e103905, doi:10.1371/journal.pone.0103905 (2014).

15 Cook, J. *et al.* Loop-mediated isothermal amplification (LAMP) for point-of-care detection of asymptomatic low-density malaria parasite carriers in Zanzibar. *Malaria Journal* **14**, 43, doi:10.1186/s12936-015-0573-y (2015).

16 Lawaly, R. *et al.* Impact of mosquito bites on asexual parasite density and gametocyte prevalence in asymptomatic chronic Plasmodium falciparum infections and correlation with IgE and IgG titers. *Infect Immun* **80**, 2240-2246, doi:10.1128/IAI.06414-11 (2012).

17 Lin, E. *et al.* Differential patterns of infection and disease with P. falciparum and P. vivax in young Papua New Guinean children. *PLoS One* **5**, e9047, doi:10.1371/journal.pone.0009047 (2010).

18 Koepfli, C. *et al.* How much remains undetected? Probability of molecular detection of human Plasmodia in the field. *PLoS One* **6**, e19010, doi:10.1371/journal.pone.0019010 (2011).

19 Wagner, G. *et al.* High incidence of asymptomatic malara infections in a birth cohort of children less than one year of age in Ghana, detected by multicopy gene polymerase chain reaction. *Am J Trop Med Hyg* **59**, 115-123 (1998).

20 Jafari-Guemouri, S., Boudin, C., Fievet, N., Ndiaye, P. & Deloron, P. Plasmodium falciparum genotype population dynamics in asymptomatic children from Senegal. *Microbes Infect* **8**, 1663-1670, doi:10.1016/j.micinf.2006.01.023 (2006).

21 Owusu-Agyei, S., Smith, T., Beck, H. P., Amenga-Etego, L. & Felger, I. Molecular epidemiology of Plasmodium falciparum infections among asymptomatic inhabitants of a holoendemic malarious area in northern Ghana. *Trop Med Int Health* **7**, 421-428 (2002).

22 Fraser-Hurt, N. *et al.* Effect of insecticide-treated bed nets on haemoglobin values, prevalence and multiplicity of infection with Plasmodium falciparum in a randomized controlled trial in Tanzania. *Transactions of the Royal Society of Tropical Medicine and Hygiene* **93 Suppl 1**, 47-51 (1999).

23 Tadesse, F. G. *et al.* The Relative Contribution of Symptomatic and Asymptomatic Plasmodium vivax and Plasmodium falciparum Infections to the Infectious Reservoir in a Low-Endemic Setting in Ethiopia. *Clinical Infectious Diseases*, cix1123-cix1123, doi:10.1093/cid/cix1123 (2018).

24 Ouedraogo, A. L. *et al.* Substantial contribution of submicroscopical Plasmodium falciparum gametocyte carriage to the infectious reservoir in an area of seasonal transmission. *PLoS One* **4**, e8410, doi:10.1371/journal.pone.0008410 (2009).

25 Gaye, A. *et al.* Infectiousness of the human population to Anopheles arabiensis by direct skin feeding in an area hypoendemic for malaria in Senegal. *Am J Trop Med Hyg* **92**, 648-652, doi:10.4269/ajtmh.14-0402 (2015).

26 Githeko, A. K. *et al.* The reservoir of Plasmodium falciparum malaria in a holoendemic area of western Kenya. *Transactions of the Royal Society of Tropical Medicine and Hygiene* **86**, 355-358 (1992).

27 Stone, W., Goncalves, B. P., Bousema, T. & Drakeley, C. Assessing the infectious reservoir of falciparum malaria: past and future. *Trends in parasitology* **31**, 287-296, doi:10.1016/j.pt.2015.04.004 (2015).

28 Coleman, R. E. *et al.* Infectivity of Asymptomatic Plasmodium-Infected Human Populations to Anopheles dirus Mosquitoes in Western Thailand. *Journal of Medical Entomology* **41**, 201-208, doi:10.1603/0022-2585-41.2.201 (2004).

29 Gonçalves, B. P. *et al.* Examining the human infectious reservoir for Plasmodium falciparum malaria in areas of differing transmission intensity. *Nature Communications* **8**, 1133, doi:10.1038/s41467-017-01270-4 (2017).

30 Okell, L. C. *et al.* Factors determining the occurrence of submicroscopic malaria infections and their relevance for control. *Nature Communications* **3**, 1237, doi:10.1038/ncomms2241 (2012).
