# Supplementary material for: The temporal dynamics and infectiousness of subpatent Plasmodium falciparum infections in relation to parasite density
Source: Nat Commun. 2019 Mar 29;10:1433. doi: 10.1038/s41467-019-09441-1 (PMC6440965; doi:10.1038/s41467-019-09441-1)
Supplement: Supplementary file 3 — Reporting Summary [file 41467_2019_9441_MOESM3_ESM.pdf]

nature research

Corresponding author(s): Hannah Slater

Last updated by author(s): Feb 14, 2019

Reporting Summary

Nature Research wishes to improve the reproducibility of the work that we publish. This form provides structure for consistency and transparency in reporting. For further information on Nature Research policies, see Authors & Referees and the Editorial Policy Checklist.

Statistics

For all statistical analyses, confirm that the following items are present in the figure legend, table legend, main text, or Methods section.

n/a	Confirmed	
☐	☒	The exact sample size (n) for each experimental group/condition, given as a discrete number and unit of measurement
☒	☐	A statement on whether measurements were taken from distinct samples or whether the same sample was measured repeatedly
☐	☒	The statistical test(s) used AND whether they are one- or two-sided<br>*Only common tests should be described solely by name; describe more complex techniques in the Methods section.*
☐	☒	A description of all covariates tested
☐	☒	A description of any assumptions or corrections, such as tests of normality and adjustment for multiple comparisons
☐	☒	A full description of the statistical parameters including central tendency (e.g. means) or other basic estimates (e.g. regression coefficient) AND variation (e.g. standard deviation) or associated estimates of uncertainty (e.g. confidence intervals)
☐	☒	For null hypothesis testing, the test statistic (e.g. F, t, r) with confidence intervals, effect sizes, degrees of freedom and P value noted<br>*Give P values as exact values whenever suitable.*
☐	☒	For Bayesian analysis, information on the choice of priors and Markov chain Monte Carlo settings
☐	☒	For hierarchical and complex designs, identification of the appropriate level for tests and full reporting of outcomes
☒	☐	Estimates of effect sizes (e.g. Cohen's d, Pearson's r), indicating how they were calculated

Our web collection on statistics for biologists contains articles on many of the points above.

Software and code

Policy information about availability of computer code

Data collection	The data collection for each study is described in each primary manuscript
Data analysis	All analyses were conducted in R using open source packages available from CRAN (https://cran.r-project.org/). Methods for the analyses are provided in the methods section or supplementary material in all instances were non-standard analyses were applied.

For manuscripts utilizing custom algorithms or software that are central to the research but not yet described in published literature, software must be made available to editors/reviewers. We strongly encourage code deposition in a community repository (e.g. GitHub). See the Nature Research guidelines for submitting code & software for further information.

Data

Policy information about availability of data

All manuscripts must include a data availability statement. This statement should provide the following information, where applicable:

- Accession codes, unique identifiers, or web links for publicly available datasets
- A list of figures that have associated raw data
- A description of any restrictions on data availability

Data availability statement
All data used in this manuscript are provided in supplementary excel spreadsheets or in online repositories previously deposited by study PI for each relevant study with original publication of data. Details of repositories are provided in the excel spreadsheets on tabs relating to the relevant study.

Field-specific reporting

Please select the one below that is the best fit for your research. If you are not sure, read the appropriate sections before making your selection.

☒ Life sciences ☐ Behavioural & social sciences ☐ Ecological, evolutionary & environmental sciences

For a reference copy of the document with all sections, see nature.com/documents/nr-reporting-summary-flat.pdf

Life sciences study design

All studies must disclose on these points even when the disclosure is negative.

Sample size	This study in a secondary analysis of >20 datasets. Sample size considerations for each study are presented in the primary manuscripts.
Data exclusions	The datasets were non-homogeneous, so various data were excluded to make them consistent. Primarily, for figures 1-3, this meant excluding febrile individuals to ensure all populations considered were just asymptomatic individuals.
Replication	All findings can be reproduced using datasets provided and methods described.
Randomization	NA
Blinding	NA

Reporting for specific materials, systems and methods

We require information from authors about some types of materials, experimental systems and methods used in many studies. Here, indicate whether each material, system or method listed is relevant to your study. If you are not sure if a list item applies to your research, read the appropriate section before selecting a response.

Materials & experimental systems

n/a	Involved in the study	
☒	☐	Antibodies
☒	☐	Eukaryotic cell lines
☒	☐	Palaeontology
☒	☐	Animals and other organisms
☐	☒	Human research participants
☒	☐	Clinical data

Methods

n/a	Involved in the study	
☒	☐	ChIP-seq
☒	☐	Flow cytometry
☒	☐	MRI-based neuroimaging

Human research participants

Policy information about studies involving human research participants

Population characteristics	The population characteristics of each study used in this secondary analysis are fully described in each of the primary manuscripts
Recruitment	The recruitment criteria of individuals in each study used in this secondary analysis are fully described in each of the primary manuscripts
Ethics oversight	Ethical approval was granted for each of the primary studies

Note that full information on the approval of the study protocol must also be provided in the manuscript.
